# Incidence of Atrial Fibrillation in Large Vessel Occlusion and Large Embolic Stroke of Undetermined Source

**DOI:** 10.7759/cureus.33700

**Published:** 2023-01-12

**Authors:** Eric J Seachrist, Ashley Petrone, Connor Nevin, Tamra Ranasinghe, Sneha Jacob, Christopher Ferari, Amelia Adcock

**Affiliations:** 1 Neurology, West Virginia University School of Medicine, Morgantown, USA; 2 Medicine, West Virginia University School of Medicine, Morgantown, USA; 3 Neurology, Wake Forest School of Medicine, Winston-Salem, USA; 4 Neurology, Wheeling Hospital, Wheeling, USA; 5 Urology, Indiana University School of Medicine, Indianapolis, USA

**Keywords:** embolic stroke, esus, large vessel occlusion, atrial fibrillation, stroke

## Abstract

Introduction: Large vessel occlusion (LVO) stroke is a common presentation of acute ischemic stroke and is often unknown or cryptogenic in etiology. There is a strong association between atrial fibrillation (AF) and cryptogenic LVO stroke, making it a unique stroke subgroup. Therefore, we propose that any LVO stroke meeting the criteria for an embolic stroke of an undetermined source (ESUS) be classified as large ESUS (LESUS). The purpose of this retrospective cohort study was to report the etiology of anterior LVO strokes that underwent endovascular thrombectomy.

Methods: This was a single-center retrospective cohort study characterizing the etiology of acute anterior circulation LVO strokes that received emergent endovascular thrombectomy from 2011 to 2018. Patients with LESUS designation at hospital discharge were changed to cardioembolic etiology if AF was discovered during the two-year follow-up period.

Results: Overall, 155 (45%) of 307 patients in the study were found to have AF. New onset AF was discovered in 12 (23%) of 53 LESUS patients after hospitalization. Furthermore, eight (35%) of 23 LESUS patients who received extended cardiac monitoring were found to have AF.

Conclusion: Nearly half the patients with LVO stroke who received endovascular thrombectomy were found to have AF. With the use of extended cardiac monitoring devices after hospitalization, AF is frequently discovered in patients with LESUS and may change the secondary stroke prevention strategy.

## Introduction

Approximately 30% of acute ischemic strokes (AIS) are large vessel occlusion (LVO) strokes, typically defined as occlusions in the proximal middle cerebral (M1), early branch middle cerebral (M2), basilar, or intracranial internal carotid arteries (ICA) [1,2]. Uniformity in LVO definition is lacking and can include proximal anterior cerebral, posterior cerebral, and vertebral artery occlusion [1]. Further, LVO stroke is associated with greater morbidity and mortality compared with a small vessel or non-LVO stroke [2]. Although the use of endovascular thrombectomy (EVT) for clot retrieval has significantly lowered mortality and improved LVO patient outcomes, the success of EVT is largely dependent on time to treatment/recanalization in the acute setting [3,4]. Therefore, early identification of LVO stroke is crucial to decreasing the time from hospital admission to treatment with EVT. Lastly, determining LVO etiology and/or identifying and mitigating risk factors for LVO stroke is crucial to prevent recurrent AIS.

Atrial fibrillation (AF) is a well-established risk factor for severe AIS and is a predictor of LVO stroke on prehospital assessment [5-7]. In five large clinical trials investigating the efficacy of EVT, approximately 33% of LVO stroke patients had known AF at the time of presentation [8]. Similarly, the prevalence of AF is doubled in patients with LVO strokes compared with all other AIS subtypes [9]. Furthermore, the cause or etiology of nearly half of all LVO cases is cardioembolism, with more than half of cardioembolic cases likely attributed to AF [10].

While a subgroup of large anterior circulation embolic strokes is attributed to known AF, up to 30% of cases still elude identification of etiology and are deemed cryptogenic [11]. Embolic stroke of undetermined source (ESUS) criteria provides a standardized definition for embolic-appearing cryptogenic strokes [12]. Additionally, ESUS exclusion criteria allow for a standardized definition of cardioembolic stroke [12]. Initial studies suggest that empiric anticoagulation after ESUS is not superior to antiplatelet therapy [12,13]; although LVO subgroup data are not known and likely underrepresented in these trials given the low median stroke scale scores. Further research on empiric anticoagulation is ongoing [14]. Recent investigations have strengthened the connection between AF and LVO [15,16]. In trials utilizing implantable and external cardiac monitors after cryptogenic stroke, the presence of LVO was the only independent predictor of AF besides age [15,16]. These findings highlight the LVO subgroup of AIS and the need for detailed occult AF investigation. Therefore, we propose that any LVO stroke meeting the criteria for ESUS be classified as a large embolic stroke of undetermined source (LESUS).

The purpose of this retrospective cohort study was to determine the prevalence of AF discovered during hospitalization and follow-up care for LVO strokes treated by EVT. We hypothesized that a significant number of LVO patients designated with LESUS etiology upon hospital discharge would be subsequently diagnosed with AF on extended cardiac monitoring during post-hospitalization follow-up care.

Some of the data in this article were previously presented as a meeting abstract at the 2019 American Academy of Neurology Annual Meeting on May 5, 2019.

## Materials and methods

We performed a retrospective review of all patients at West Virginia University who received emergent EVT for acute anterior circulation LVO stroke. This project was approved by the institutional review board (approval no. 1607198959), and individual patient consent was waived under the common rule/waiver of authorization. This study includes all items that should be included in observational studies as per the strengthening of the reporting of observational studies in epidemiology (STROBE) checklist for cohort studies [17].

Inclusion criteria consisted of presentation to the emergency department of West Virginia University for stroke, presence of LVO as defined below, attempted treatment with EVT, and age 18 years or greater. Included patients are those who presented for EVT from January 2011 to December 2018. Follow-up and post-hospitalization data were included for LESUS patients up to two years after EVT. For this study, anterior LVO stroke was defined as an occlusion of either the intracranial ICA, proximal middle cerebral artery (M1), or tandem ICA and M1 occlusions. Early branch middle cerebral artery (M2), proximal anterior cerebral artery, and posterior circulation occlusions were excluded due to a lack of uniformity in thrombectomy treatment. All patients received computed tomography angiography (CTA) to confirm LVO and subsequently met a neuroradiologist’s recommendation to receive EVT. Patients who received EVT had a large ischemic penumbra with a small core infarct on computed tomography perfusion studies and were last seen normal within 24 hours of presentation, although the vast majority were treated within six hours of last seen normal.

Cases were reviewed and classified for stroke etiology similar to TOAST criteria [18]: large-artery disease (LAD); cardioembolism; stroke of other determined sources including hypercoagulable state, patent foramen ovale (PFO), and cerebral vasculitis; and stroke of undetermined etiology. Given that only LVO strokes were included in this study, no patients had small-vessel occlusion. Similarly, all strokes classified as strokes of undetermined etiology met the criteria for ESUS and therefore, LESUS. The ESUS criteria included non-lacunar stroke, less than 50% ipsilateral intra or extracranial artery stenosis, no high risk for cardioembolism, and no other source of strokes such as artery dissection, vasospasm, illicit drug use, or arteritis [12]. Standard hospitalization workup to determine etiology included vascular imaging, echocardiogram by transthoracic and/or transesophageal methods, electrocardiogram, and continuous telemetry monitoring. Since all patients received EVT, direct catheter angiography was used as the vascular imaging of choice for the determination of vessel stenosis severity. Testing for hypercoagulable disorders was performed only in select cases, typically young patients with minimal atherosclerotic disease and no other source of stroke, as per guidelines [19]. Hypercoagulable etiology included active systemic malignancy, positive hypercoagulable blood tests, or similar conditions treated with chronic anticoagulation. As cardiovascular risk factors, we included known obstructive sleep apnea and the presence of left atrial enlargement on echocardiogram. 

Our etiology classification system differed from TOAST criteria on several points. Cardioembolic etiology was defined by the presence of one or more ESUS exclusion criteria: AF, left ventricular thrombus, mechanical heart valve, severe mitral stenosis, myocardial infarction less than four weeks from presentation, ejection fraction less than 30%, or endocarditis [12]. The PFO strokes were placed under the stroke of determined source category rather than cardioembolic, given its unique diagnosis criteria and treatment. Etiology was determined to be PFO only after a complete workup, including extended cardiac monitoring, was negative, as per guidelines [20]. Large-artery disease included patients with ipsilateral carotid stenosis greater than 50%, acute carotid dissection, or significant intracranial stenosis of the ipsilateral middle cerebral artery. Given the inclusion of dissection, we termed this group LAD rather than large-artery atherosclerosis. Additionally, all tandem occlusions were classified as LAD for uniformity and given their typical atherothrombotic or dissection mechanism. Lastly, we followed ESUS guidelines for a stroke of an undetermined source to categorize patients with multiple stroke etiologies. For example, if a patient had both newly discovered AF and significant ipsilateral cervical internal carotid artery stenosis, etiology was deemed LAD for uniformity. Patients with no known stroke cause after discharge were deemed LESUS, including those with early mortality or incomplete standard workup.

Except in cases of early mortality or rare lack of follow-up, LESUS patients received follow-up care up to two years from the date of LVO by either outpatient neurology assessment or repeat hospitalization. Some received extended cardiac monitoring devices, including a 30-day event monitor and/or implantable loop recorder, with recording up to 12 months from loop recorder implantation. Other LESUS patients received repeat electrocardiograms at outpatient follow-up or repeat hospitalization. 

Statistical analysis was performed using SSPS software, version 28.0 (IBM Corp., Armonk, NY, USA). Frequency analysis was utilized as a descriptive method to determine the frequency or proportion of each study variable in our patient sample. For continuous variables, normality was assessed using the Shapiro-Wilk test, and either mean or median was reported, as appropriate.

## Results

Three hundred and seven patients who received EVT for the treatment of anterior circulation LVO were included. The median age of the study population was 70 years, and males and females were represented equally. The prevalence of known AF at the time of admission was 33%. The median National Institutes of Health Stroke Scale (NIHSS) upon presentation to the hospital was 17 (Table 1). The LVO occlusion was further divided by location: 74% of cases were M1, 14% were ICA, and 12% were tandem occlusions (Table 2).

**Table 1:** Study population demographics IV tPA: Intravenous tissue plasminogen activator

Total Population (number)	307
Age (median years)	70
Female (%)	50
Known Atrial Fibrillation (%)	33
Admission NIHSS (median score)	17
Received IV tPA (%)	49

**Table 2:** Percentage of each type of LVO occlusion This table lists each subtype of LVO stroke based on the location of the occlusion. These location types are proximal middle cerebral artery, M1, distal ICA, and tandem, which are the combined occlusions of the cervical ICA and proximal middle cerebral artery. The M1 was the most common location for LVO and accounted for 74% of all LVO strokes in the study. LVO: Large vessel occlusion, ICA: Internal carotid artery

LVO Type	Percentage
M1	74
ICA	14
Tandem	12

At hospital discharge, 45% of LVO patients had either known or newly discovered AF. This included the previous diagnosis of AF, AF discovered on initial electrocardiogram, and AF discovered on subsequent inpatient cardiac monitoring. Stroke etiology at time of hospital discharge: 50% cardioembolic including those with AF, 20% LAD, 5% other determined sources (six PFO, 10 hypercoagulable conditions, and one cerebral vasculitis), and the remaining 25% of cases were considered LESUS (Table 3).

**Table 3:** LVO etiology at hospital discharge and after follow up This table shows percentages of each stroke etiology before and after the follow-up period. Other known etiology includes strokes due to patent foramen ovale, hypercoagulable, conditions, and cerebral vasculitis. LVO: Large vessel occlusion

Etiology	Percentage at Discharge	Percentage After Follow-up
Large Artery Disease	20	20
Cardioembolic	50	54
Other Known Etiology	5	5
Large Embolic Stroke of Undetermined Source	25	21

At the time of hospital discharge, 76 patients (25%) were classified as LESUS. Among them, 23 were excluded from further analysis due to lack of follow-up (two), death during hospitalization or shortly after discharge (13), use of anticoagulation (six), and refusal of extended cardiac monitoring (two). Of the remaining 53 LESUS patients, new-onset AF was discovered in 12 (23%) patients: eight were found to have AF on extended cardiac monitoring devices, and four were found to have AF on routine electrocardiogram. Patients with newly discovered AF during the follow-up period were reclassified from LESUS to cardioembolic etiology (as seen above in Table 3). The demographics and risk factors of these 12 patients did not greatly differ from the LESUS patients not found to have AF. Only two of these patients had obstructive sleep apnea, two had left atrial enlargement on echocardiogram, and one was less than 60 years old. Nine of these patients had M1 occlusions, while the remaining three had distal ICA occlusions. 

Only 23 of 53 eligible LESUS patients received extended cardiac monitoring devices. Reasons for the lack of extended cardiac monitoring device use included early mortality after discharge, anticoagulation use, not recommended by treating neurologist, and patient refusal. Thirty-day event monitor was the most frequently utilized cardiac monitoring device as implantable loop recorder use was infrequent overall. Of the eight LESUS patients with AF discovered by extended cardiac monitoring, a 30-day event monitor was used in seven, while an implantable loop recorder was used in one. Only five LESUS patients who did not have AF discovered by the 30-day event monitor received an implantable loop recorder for further cardiac monitoring, and one of these patients had AF discovered. 

Throughout the study period, AF was found in 50% of patients who presented for EVT of anterior LVO stroke. Of the 138 patients diagnosed with AF, 66% of AF cases were known prior to admission or were found on initial electrocardiogram prior to EVT, 24% were discovered by subsequent cardiac monitoring during hospitalization, and 10% during routine follow-up from LVO hospitalization.

## Discussion

A large proportion of anterior circulation LVO strokes treated with EVT is attributed to a cardioembolic source, which in most cases is AF. Our study aimed to identify rates of AF among LVO strokes treated with EVT and to propose a subset of patients at particularly high risk for AF, termed LESUS. Approximately 33% of LVO strokes in the large randomized EVT clinical trials had AF on presentation, although these studies did not account for AF detected during or after hospitalization [8]. This was also observed in our sample as 33% (102/307) of patients had known AF at the time of LVO stroke. Ultimately, more than half (54%) of all LVO strokes were classified as cardioembolic; furthermore, most of these (84%) had underlying AF. Large embolic stroke of an undetermined source was the second most common etiology at the time of hospital discharge (25%), which reflects historical rates of cryptogenic LVO [11,21]; although, 4% of these LESUS patients were reclassified as cardioembolic etiology after AF discovery.

As hypothesized, a high proportion (23%) of applicable patients with LESUS were found to have atrial fibrillation after discharge. We suspect that far more patients would have had AF detected, but only 43% of applicable patients with LESUS underwent extended cardiac monitoring during the follow-up period. As recently reported, the presence or absence of LVO was the only statistically significant predictor of AF on 28-day Holter monitoring, apart from the well-established association with age [15]. In the cryptogenic stroke and underlying AF trial, AF was detected in 8.9% and 12.4% at six months and 12 months, respectively [22]. These trial patients included a younger and more heterogenous stroke population than in our study. Current guidelines support cardiac rhythm monitoring after stroke although the duration and prioritization among stroke subgroups are unclear [23]. In our study, 35% of LESUS patients who underwent extended cardiac monitoring for one to 12 months duration were found to have AF. Similarly in a recent study, AF was found by implantable cardiac monitoring devices in 41% of cryptogenic LVO stroke patients compared to 13% of cryptogenic non-LVO patients [16]. This further supports the high likelihood of an underlying AF-related cardioembolic etiology among LESUS patients and the utility of extended monitoring in this stroke subgroup.

The discovery of AF typically guides secondary stroke prevention management toward anticoagulation rather than antiplatelet therapies [24]. Standardized rhythm monitoring protocols post-stroke are yet to be established. Typical approaches vary widely from a 24-hour Holter monitor to implantable loop recorders lasting for years in duration. The ESUS trial inclusion criteria require at least 20 hours of cardiac monitoring to exclude AF before ESUS classification [12,13]. Considering multiple studies demonstrating improved AF identification using extended cardiac monitoring among cryptogenic stroke patients, 20 hours is unlikely to be adequate to diagnose AF post-stroke [25,26]. The benefit of prolonged monitoring is reflected in current guidelines, listing implantable cardiac recorders as reasonable after stroke [24]. A recent study found a high incidence of AF when implantable cardiac monitoring was used after both large and small vessel strokes [27]. Although AF after stroke may not indicate direct causation, it is likely at least a marker of left atrial dysfunction and cardioembolic risk [28]. While the clinical meaningfulness of incidentally discovered AF by implantable cardiac monitoring after non-cardioembolic stroke is unclear, AF discovery after a correlating stroke event, such as LESUS, confers high clinical significance. The presence of AF after stroke typically necessitates anticoagulation treatment by risk scoring calculation [24].

Atrial fibrillation discovery after LESUS, therefore, will alter secondary stroke prevention strategies. In certain cases, empiric anticoagulation can be considered although it remains controversial. In two large clinical trials, empiric anticoagulation was tested among general ESUS patients and was not beneficial [12,13]. However, these studies were designed to exclude patients who were at a high risk of AF and generally had lower vascular risk factor profiles. In Navigate ESUS, only 7% of patients had a history of coronary artery disease [29]. In RE-SPECT ESUS, subgroup analysis demonstrated anticoagulation benefits in patients with risk factors for AF: older than 75, higher CHADs2-VASc scores, and less baseline cardiac monitoring [13]. Rather than recommending anticoagulation to the heterogenous ESUS population, a more focused approach toward patients who are at high risk for underlying cardioembolic etiology with established anticoagulation benefits, like AF, would better guide treatment [30]. This study suggests that LVO strokes of an underdetermined source, LESUS, represent one of these high-risk AF subgroups and be considered in future studies of empiric anticoagulation.

This study has several limitations in addition to those inherent to any retrospective review. While the generalization is limited to a single institution study, our rates of AF with LVO stroke are similar to multicenter and international trials. It is possible that the population of LVO patients who undergo EVT is biologically different than those who do not receive this treatment. For example, an acute embolic process like AF may be more likely to present in time for EVT as opposed to a more subacute process like intracranial atherosclerosis. The lack of LVO strokes without endovascular intervention is a systemic challenge as most LVO studies focus on treated populations. Although the authors determined the most probable stroke etiology based on a systemic chart review and rigorously defined criteria, misclassification may have occurred. In times of ambiguity or dual mechanisms, priority was given to LAD which may have underestimated the cardioembolic source.

A final limitation is the inconsistent use of long-term cardiac monitoring. Less than half (43%) of LESUS patients appropriate for detailed cardiac rhythm investigation received it, probably underestimating the true proportion of LESUS patients with occult AF. Additionally, few patients received implantable loop recorders. Compliance with complicated insurance approvals for implantable recorders or cumbersome monitoring devices has been associated with poor adoption of guidelines in clinical practice [27,28], although only two of our patients deferred monitoring. These drawbacks may be overcome by streamlining operations and initiating monitors upon stroke discharge.

## Conclusions

More than half of LVO strokes undergoing EVT are due to a cardioembolic source, specifically AF. With the use of extended cardiac monitoring devices, AF is frequently discovered in patients with large embolic stroke of undetermined source (LESUS). The identification of AF after stroke typically changes the treatment strategy for secondary stroke prevention. The use of extended cardiac monitoring for AF, therefore, is critical in the evaluation of LESUS strokes. Further studies should consider if the LESUS subgroup would benefit from empiric anticoagulation.
